# The role of structured remembrance programs in supporting the wellbeing of pediatric health workers

**DOI:** 10.1017/S1478951524001949

**Published:** 2025-01-21

**Authors:** Rikas Saputra, Isnaria Rizki Hayati, Elni Yakub, Yenni Lidyawati, Rizky Andana Pohan, Mei Sarri

**Affiliations:** 1 Department of Islamic Guidance and Counselling, Universitas Islam Negeri Raden Fatah Palembang, Palembang, South Sumatra, Indonesia; 2 Department of Guidance and Counselling, Universitas Riau, Pekanbaru, Indonesia; 3 Department Indonesian Language and Literature Education, Universitas Sriwijaya, Palembang, Indonesia; 4 Department of Islamic Guidance and Counseling, Institut Agama Islam Negeri Langsa, Kota Langsa, Aceh, Indonesia; 5 Department of Guidance and Counselling, Universitas Negeri Semarang, Central Java, Indonesia

Dear Editor,

We would like to submit a response to the article “Making Space for Grief: The Impact of Remembrance Programmes on Paediatric Healthcare Providers” by Wiener and Ph (2024). Through a structured remembrance program, the authors have presented significant insights into grief support for healthcare workers in pediatric units. As revealed by the authors, the grief experienced by healthcare workers when caring for a deceased pediatric patient is profound, affecting their mental well-being and overall job satisfaction. Programs such as the *Pediatric Remembrance Ceremony* (PRC) (Pang 2024) and *Good Grief and Chocolate at Noon* (GGCN) provide much-needed space for reflection and mutual support, as well as an approach to dealing with the emotional burden of pediatric care.

Rabow et al. (2021) highlighted the importance of candle lighting and name reading in strengthening emotional bonds between health workers. PRCs, structured yet personalized, allow staff to honor the departed patient while acknowledging their shared grief (Butler et al. 2024). This strengthens solidarity while reducing feelings of alienation among key staff in overcoming burnout and *compassion fatigue* (Kinsella et al. 2023). The article underlines that such rituals can provide emotional relief, reinforcing the idea that structured remembrance practices can be therapeutic in medical environments, where grief experiences are often hidden or rarely openly expressed (Maddrell 2016).

This study also highlights the unique format of the GGCN program, which allows for informal exchange and sharing of personal stories. Such programs fill a void in formal grief support by offering the opportunity for authentic, spontaneous sharing among colleagues (Testoni et al. 2023). By creating a space for healthcare workers to openly share their personal experiences and listen to the stories of others, GGCN creates an environment that supports the growth of empathy and understanding. As noted by the authors, the transition to a virtual format due to the COVID-19 pandemic brought unexpected benefits, such as enabling more comprehensive access while maintaining emotional safety (Barreda-Ángeles and Hartmann 2022). This may encourage more frequent participation, as grieving privately through a computer screen may feel more comfortable for some (Entilli et al. 2024).

However, one aspect that needs further research is the long-term impact of these memorial services on the psychological well-being of health workers (Ali et al. 2021). Although these programs offer immediate emotional relief, long-term evaluations may provide insights into whether continued participation contributes to resilience against burnout (Archer et al. 2024). Integrating additional strategies, such as regular follow-up sessions, may amplify the cumulative benefits, ensuring that these efforts match staff needs (Wright et al. 2023).

The authors’ recommendations for a hybrid model are well suited to the current work environment and can serve as a reference for other institutions (Naqshbandi et al. 2024). By combining virtual and face-to-face options, these remembrance programs have the potential to expand reach, offering grief support tailored to individual preferences and convenience (Bott et al. 2019). This approach provides an opportunity to improve the mental well-being of healthcare workers and the quality of patient care they provide (De Kock et al. 2021). Implementing this hybrid model across different healthcare settings may boost staff morale and contribute to an empathetic work culture (Sampat et al. 2022).

Remembrance programs such as PRC and GGCN have great potential in supporting healthcare workers facing bereavement in pediatric care units. Their continued implementation, with further studies to measure their impact, could provide a sustainable framework for healthcare systems worldwide to support healthcare workers in dealing effectively with bereavement.
